# Association Between Type 2 Diabetes Mellitus and Sepsis Outcomes: A Systematic Review and Meta-Analysis

**DOI:** 10.7759/cureus.111148

**Published:** 2026-06-19

**Authors:** Navya Reddy Gade, Rohit R Dugyala, Tulasi Nandam, Sreeja Kommalapati, Sudeep Aitham, Harinisree Keerthipati

**Affiliations:** 1 Department of Endocrinology, Gandhi Medical College and Hospital, Secunderabad, IND; 2 Department of Internal Medicine, Gandhi Medical College and Hospital, Secunderabad, IND

**Keywords:** mortality, random-effects model, sepsis, systematic review and meta-analysis, type 2 diabetes mellitus

## Abstract

Sepsis is a major cause of morbidity and mortality worldwide. Type 2 diabetes mellitus (T2DM) is common among adults with infection and has been proposed as a potential modifier of sepsis outcomes. This systematic review and meta-analysis aimed to evaluate the association between pre-existing T2DM and mortality among adults with sepsis. This review was conducted in accordance with the Preferred Reporting Items for Systematic Reviews and Meta-Analyses 2020 guidelines. PubMed, Cochrane CENTRAL, and Google Scholar databases were systematically searched from inception to April 23, 2026. Observational studies comparing outcomes in adult patients with sepsis with and without T2DM were eligible. Pooled odds ratios (ORs) with 95% confidence intervals (CIs) were calculated using a random-effects model. A total of 651 records were identified. Seven studies were included in the qualitative synthesis, and three in the primary quantitative meta-analysis. The pooled analysis showed no statistically significant association between T2DM and sepsis mortality (OR = 0.99; 95% CI = 0.82-1.20). However, substantial heterogeneity was observed across studies (I² = 98.4%). Sensitivity and exploratory analyses yielded similar findings. Narrative evidence suggested that glycemic status, diabetes-related complications, and acute renal dysfunction may be more important determinants of outcomes than the diagnosis of T2DM alone. The current evidence did not demonstrate a statistically significant association between pre-existing T2DM and mortality among adults with sepsis. However, these findings should be interpreted cautiously given the limited number of studies included in the primary meta-analysis, the substantial heterogeneity across studies, and the observational nature of the available evidence. Future prospective, well-adjusted studies should evaluate the impact of glycemic control, diabetes-related complications, organ dysfunction, and antidiabetic treatment exposure on sepsis outcomes.

## Introduction and background

Sepsis is a life-threatening syndrome characterized by acute organ dysfunction caused by a dysregulated host response to infection [1]. It remains a significant global health problem despite advances in critical care and the implementation of standard management practices, leading to prolonged hospitalization and post-discharge complications [2,3]. Meanwhile, the prevalence of type 2 diabetes mellitus (T2DM) has increased dramatically, with hundreds of millions of people living with diabetes worldwide, the majority of whom have T2DM [4]. Individuals living with diabetes are more susceptible to infections and are at a higher risk of hospitalization due to infectious diseases such as pneumonia, soft-tissue infections, and bloodstream infections [5,6].

The causes of this increased vulnerability are complex. Host defense and organ dysfunction in infection may be caused by hyperglycemia, insulin resistance, endothelial dysfunction, and immune dysfunction, including neutrophil dysfunction and cytokine dysregulation [7-10]. However, the relationship between T2DM and sepsis outcomes remains unclear. The prior observational studies have not been consistent, with some showing no difference/or even lower mortality among individuals with diabetes and other conditions that are related to amplified mortality, especially in patients with severe complications of diabetes or organ dysfunction [11-15]. More recent evidence indicates that the presence of diabetes is not the sole factor that may predict outcomes in patients with sepsis, and that glucose control, variability in blood glucose, complications, and antidiabetic medication use may be more crucial [16-20]. Against this backdrop of these competing accounts and the newfound insights into the pathophysiology of diabetes, a more specific synthesis of evidence is needed to examine the role of T2DM (as opposed to a heterogeneous population of diabetes) as an exposure. Therefore, the present study aims to survey and meta-analyze the evidence on the role of T2DM on clinical outcomes, such as mortality, in adults with sepsis. We also evaluate how T2DM affects organ dysfunction, healthcare resource utilization, and other factors that may influence prognosis.

## Review

Methodology

Design and Reporting

The 2020 Preferred Reporting Items of Systematic Reviews and Meta-Analyses (PRISMA) declaration was followed in conducting this meta-analysis and systematic review [21]. No protocol was prospectively registered, and retrospective registration was not performed because the review had already been completed. This is acknowledged as a limitation of the study.

Information Sources and Search Strategy

A systematic literature search was conducted in PubMed, Cochrane CENTRAL, and Google Scholar (via Publish or Perish) from database inception to April 23, 2026. The search strategy combined controlled vocabulary and free-text terms related to sepsis, T2DM, and clinical outcomes using Boolean operators.

For Google Scholar searches conducted through Publish or Perish, the first 200 records ranked by relevance were exported for screening. This approach was adopted because Google Scholar retrieves a very large number of citations, and the relevance of results generally declines beyond the highest-ranked records. Screening all retrieved records was not feasible, and limiting screening to the first 200 results is a commonly used pragmatic approach in systematic reviews utilizing Google Scholar. Relevance ranking was determined automatically by the Google Scholar algorithm and accessed through Publish or Perish. To minimize the risk of missing eligible studies, supplementary searches were conducted in PubMed and Cochrane CENTRAL, and the reference lists of all included studies were screened manually. Database-specific search strategies and search yields are summarized in Table 1.

**Table 1:** Database yield and electronic search strategies.

Database	Search strategy	Records identified
PubMed	(“Sepsis”[Mesh] OR sepsis[tiab] OR “septic shock”[tiab] OR “severe sepsis”[tiab]) AND (“Diabetes Mellitus, Type 2”[Mesh] OR “type 2 diabetes mellitus”[tiab] OR “type 2 diabetes”[tiab] OR T2DM[tiab]) AND (mortality[tiab] OR outcome*[tiab] OR prognosis[tiab] OR survival[tiab] OR “clinical outcome*”[tiab] OR “in-hospital mortality”[tiab] OR “ICU mortality”[tiab] OR “length of stay”[tiab] OR “organ failure”[tiab] OR “mechanical ventilation”[tiab])	434
Cochrane CENTRAL	(sepsis OR “septic shock” OR “severe sepsis”) AND (“type 2 diabetes mellitus” OR “type 2 diabetes” OR T2DM) AND (mortality OR prognosis OR survival)	17
Google Scholar/Publish or Perish	sepsis AND (“type 2 diabetes mellitus” OR “type 2 diabetes” OR T2DM) AND (mortality OR outcomes OR prognosis OR survival)	200

Eligibility Criteria

Inclusion criteria: Studies were included if they met all predefined eligibility criteria. Eligible studies included adult patients with sepsis, severe sepsis, or septic shock. T2DM had to be the primary exposure of interest or a well-defined subgroup. Studies also had to have a comparator group of patients without diabetes or without T2DM. Further, studies had to report mortality or other relevant clinical outcomes and be eligible. Only observational studies (cohort and case-control) were included.

Exclusion criteria: Studies were excluded if they were reviews, editorials, letters, commentaries, animal studies, or case reports. Conference abstracts were also excluded if they did not contain usable outcome data. Mechanistic studies without a clinical outcome comparison were excluded. Studies were also excluded if they did not clearly define diabetes exposure groups or did not have an appropriate comparator group. Duplicate or overlapping reports were removed, except when one report provided the most complete or relevant data for analysis.

Data Extraction and Study Selection

All search outcomes were combined, and the duplicates were removed. Two stages were used to filter the studies: a full-text review of the abstracts and titles of possibly relevant studies. The extracted data included author details, publication year, nation, research design, environment, sample size, number of T2DM patients, comparator, mortality outcome, effect estimate, and outcomes of organ dysfunction or healthcare use. Where multiple effect estimates were reported, the most adjusted and relevant effect estimate was selected to be synthesized. Research that did not fit the requirements to be included in the primary pooled analysis because of relevant mortality outcomes was included in a qualitative synthesis or sensitivity study.

Risk-of-Bias Assessment

The Newcastle-Ottawa Scale was utilized to evaluate the quality of non-randomized studies [22]. The scale assessed the research in the areas of outcome, comparability, and selection. High-quality studies had a score of 8-9, while moderate-quality studies received a score of 6-7.

Statistical Analysis

The main outcome was death in adults with sepsis and T2DM versus non-diabetic patients. Because clinical and methodological variability was anticipated among the studies included, odds ratios (ORs) with 95% confidence intervals (CIs) were pooled using a random-effects model [23]. The I² statistic was used to assess statistical heterogeneity [24]. The primary meta-analysis comprised studies that provided ORs for well-defined T2DM groups. Additional analyses included a sensitivity analysis using ORs with one extra study reporting crude mortality data and an exploratory relative-effect analysis including one study with an adjusted hazard ratio. Funnel plot analysis was not conducted due to the limited number of studies (<10) for the pooled mortality outcome [25].

Results

Study Selection

**Figure 1:**
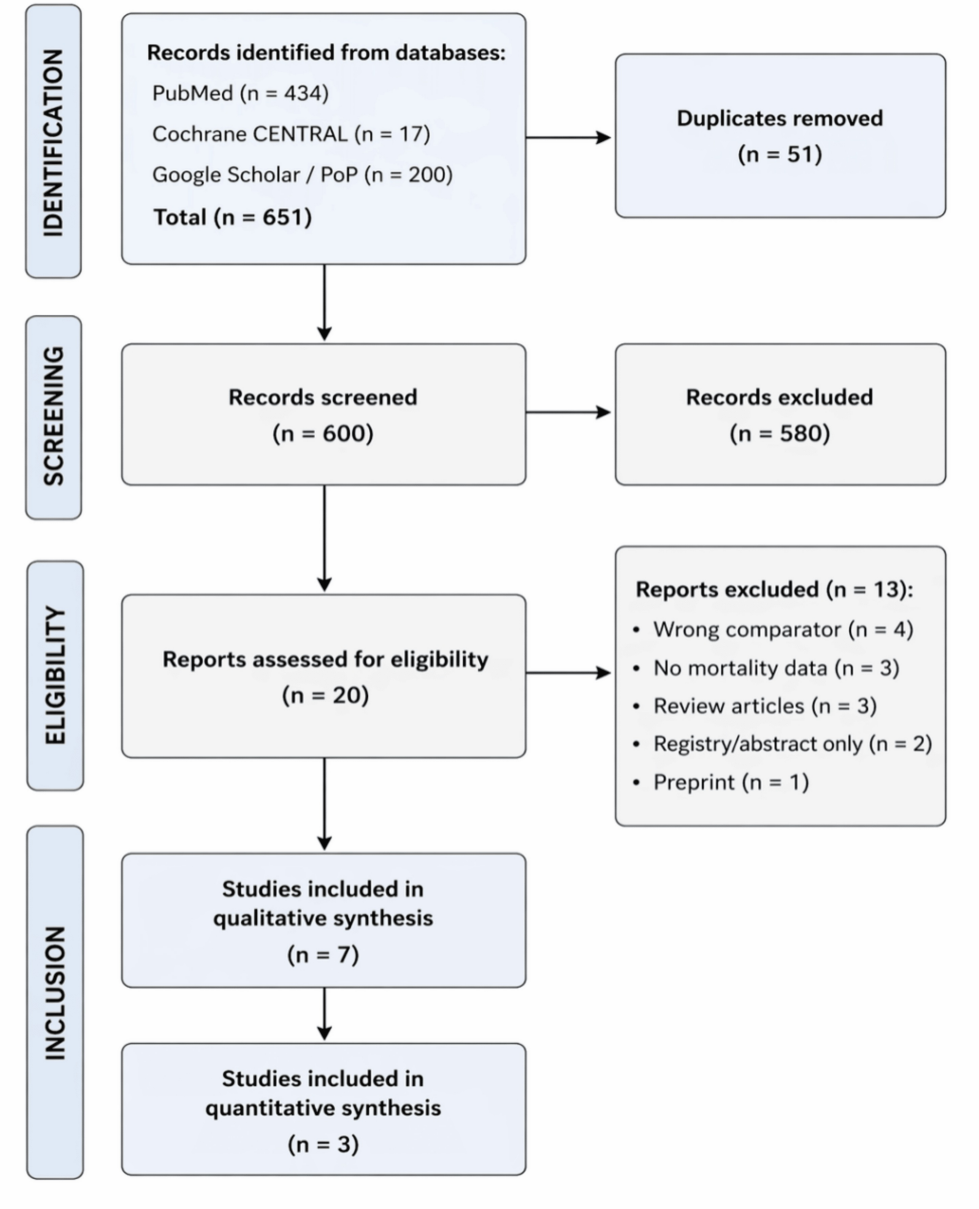
Preferred Reporting Items of Systematic Reviews and Meta-Analyses (PRISMA) flow diagram.

A total of 651 records were identified in the database search, of which 434 were from PubMed, 17 from Cochrane CENTRAL, and 200 from Google Scholar/Publish or Perish. In total, 51 duplicates were removed, leaving 600 records to be screened by title and abstract, from which 580 were excluded. The remaining 20 full-text articles were assessed for eligibility. Finally, seven studies were included in the qualitative synthesis and three studies in the main quantitative meta-analysis. The complete study selection process is presented in Figure 1.

Study Characteristics

Overall, three studies were included in the primary pooled analysis because they reported adjusted ORs for mortality in clearly defined T2DM populations. Sathananthan et al. [16] and Akinosoglou et al. [17] were not included because adjusted effect estimates compatible with the primary analysis were not available. The study by Zohar et al. [18] reported adjusted hazard ratios rather than ORs and was included only in the exploratory analysis. Han and Fang [19] reported only crude mortality estimates and were therefore included in the sensitivity analysis rather than the primary meta-analysis. Consequently, the remaining studies were incorporated into the qualitative synthesis, sensitivity analysis, or exploratory analysis according to the effect measures reported [16-19] (Table 2).

**Table 2:** Features of the studies included in the qualitative synthesis. T2DM = type 2 diabetes mellitus; ICU = intensive care unit; OR = odds ratio; HR = hazard ratio

Study	Country	Design	Setting	Total (n)	T2DM (n)	Non-DM (n)	Mortality outcome	Effect measure
Chang et al. (2012) [13]	Taiwan	Retrospective cohort	ICU	16,497	4,573	11,924	90-day mortality	Adjusted OR
de Miguel-Yanes et al. (2015) [14]	Spain	Retrospective cohort	Hospital	217,280	50,611	166,669	In-hospital mortality	Adjusted OR
Hsieh et al. (2019) [15]	Taiwan	Propensity-matched cohort	Hospital	39,438	19,719	19,719	Hospital mortality	OR
Sathananthan et al. (2020) [16]	USA	Retrospective cohort	ICU	1,698	508	1,190	30-day mortality	Not poolable
Akinosoglou et al. (2021) [17]	Greece	Cohort (registry-based)	Non-ICU	812	406	406	28-day mortality	Not poolable
Han and Fang (2025) [19]	China	Retrospective cohort	Hospital	256	151	105	In-hospital mortality	Crude OR
Zohar et al. (2021) [18]	Israel	Retrospective cohort	Hospital	1,527	469	1,058	In-hospital/90-day mortality	Adjusted HR

Risk-of-Bias Assessment

The Newcastle-Ottawa Scale was used to evaluate the methodological quality of the included studies. The scores were between 6 and 8, which is considered moderate-to-high study quality. The quality of all studies included in the primary meta-analysis was high (8). The other studies were rated as moderate-to-high quality. The most common issues raised in the studies were exposure definition, residual confounding, and outcome measurement. The results of the risk-of-bias assessment are summarized in Table 3.

**Table 3:** Evaluation of included studies using the Newcastle-Ottawa Scale.

Study	Selection	Comparability	Outcome	Total	Quality category
Chang et al. (2012) [13]	4	2	2	8	High
de Miguel-Yanes et al. (2015) [14]	4	2	2	8	High
Hsieh et al. (2019) [15]	4	2	2	8	High
Sathananthan et al. (2020) [16]	3	2	2	7	Moderate
Akinosoglou et al. (2021) [17]	4	2	2	8	High
Han and Fang (2025) [19]	3	1	2	6	Moderate
Zohar et al. (2021) [18]	3	2	2	7	Moderate

Quantitative Synthesis: Primary Outcome of Mortality

The primary meta-analysis included three studies that reported adjusted mortality estimates as ORs. The pooled analysis using a random-effects model revealed no significant association between T2DM and mortality in adults with sepsis. The pooled OR was 0.99 with a 95% CI of 0.82 to 1.20. There was significant heterogeneity between the studies (I² = 98.4%), suggesting that there was a high degree of variation in the effect estimates reported. The substantial heterogeneity observed may reflect important clinical and methodological differences among the included studies, including variations in study settings, sepsis severity, mortality endpoints, and adjustment methods. Therefore, the pooled estimate should be interpreted with caution.

**Figure 2:**
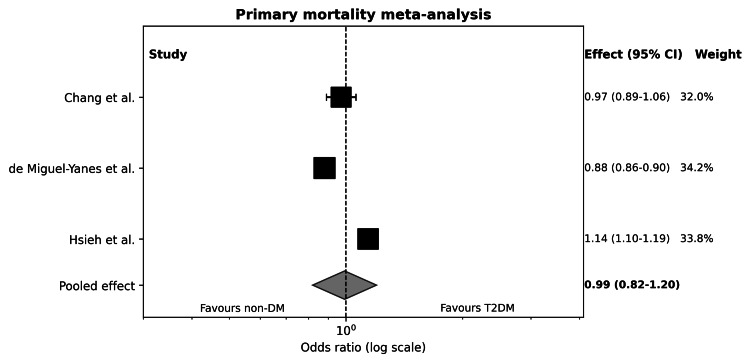
Forest plot of primary mortality meta-analysis. T2DM = type 2 diabetes mellitus; CI = confidence interval

The forest plot in Figure 2 illustrates how T2DM affects adult sepsis mortality. Each study has its estimates in the form of squares. The horizontal lines represent 95% CIs, and their sizes correspond to the study’s weight. The pooled effect, which the random-effects model estimates, is represented by the diamond.

The OR was 0.99 (95% CI = 0.82-1.20), which showed that T2DM and mortality did not significantly correlate. Studies were very heterogeneous (I² = 98.4), and study effect estimates were both negative and positive and differed in magnitude.

Sensitivity and exploratory analyses were performed. An OR-only sensitivity analysis including one additional study with crude mortality data yielded a pooled OR of 1.04, with a 95% CI of 0.86-1.25 and an I² of 97.7%. An exploratory analysis incorporating one study reporting hazard ratios showed a relative effect of 1.02, with a 95% CI of 0.85-1.21 and an I² of 97.6%.

**Figure 3:**
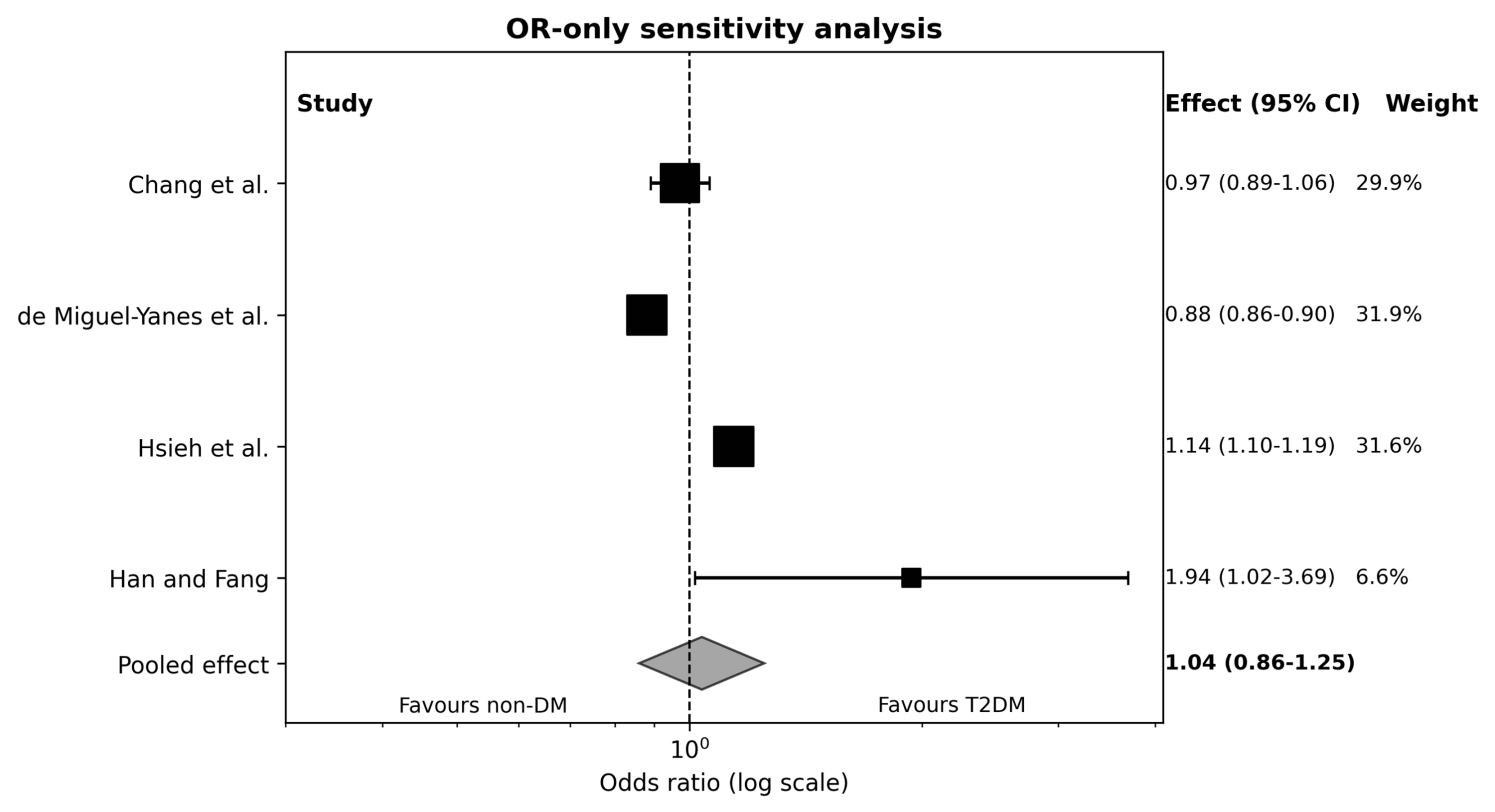
Sensitivity analysis (OR-only) forest plot. T2DM = type 2 diabetes mellitus; OR = odds ratio; CI = confidence interval

The results of the sensitivity analysis of four studies whose ORs of mortality in adults with sepsis are compatible are presented in Figure 3. The squares of each study with estimated 95% CIs are displayed by the horizontal lines and the size of the squares, which correlate to the weight. The pooled estimate is represented by a diamond (random-effects model).

The OR was 1.04 (95% CI = 0.86-1.25), demonstrating that T2DM was not significantly related to mortality. Inclusion of a single study that had crude mortality estimates did not alter the estimate. They still had considerable heterogeneity (I² = 97.7).

Qualitative Findings

Additional information was provided by other studies that were not part of the main meta-analysis. Even though no significant differences in mortality were reported between non-diabetic and diabetic patients, some studies indicated a greater occurrence of severe kidney damage and renal replacement treatment in T2DM patients. According to other studies, HbA1c parameters, hyperglycemia, and glucose variability were related to poor sepsis outcomes. Moreover, the pattern of organ dysfunction was different. A summary of the quantitative, exploratory, and qualitative syntheses findings is presented in Table 4.

**Table 4:** Summary of quantitative and qualitative findings. OR = odds ratio

Outcome	Studies (n)	Effect estimate	I² (%)	Interpretation
Primary meta-analysis	3	OR = 0.99 (0.82–1.20)	98.4	No significant association
OR-only sensitivity	4	OR = 1.04 (0.86–1.25)	97.7	No change in conclusion
Exploratory analysis	4	OR = 1.02 (0.85–1.21)	97.6	Consistent findings
Qualitative evidence	2	Not pooled	—	Mixed clinical outcomes

Discussion

The statistical significance of T2DM and mortality in persons with sepsis was not determined in this meta-analysis and systematic review. The general estimate was close to the null (OR = 0.99), indicating that T2DM is not significantly related to mortality. However, the analysis showed considerable heterogeneity, i.e., there was a high chance that the results would not be similar across various studies and clinical conditions. The substantial heterogeneity observed (I² = 98.4%) likely reflects important differences across the included studies. These studies were conducted in diverse settings, including intensive care units (ICUs), non-ICUs, and general hospital populations, and evaluated different mortality endpoints such as in-hospital, 28-day, 30-day, and 90-day mortality. Variations in sepsis severity, study design, geographic region, definitions of T2DM, adjustment variables, and baseline comorbidities may also have influenced the reported effect estimates. Given these clinical and methodological differences, the pooled estimate should be interpreted cautiously and viewed as an overall summary of the available evidence rather than a uniform effect across all patient populations.

Based on the available evidence, no significant association between T2DM and mortality was observed in adults with sepsis. However, given the limited number of studies included in the primary meta-analysis, the substantial heterogeneity, and the observational nature of the evidence, these findings should be interpreted cautiously. Further prospective studies are needed to clarify the relationship between T2DM and sepsis outcomes. Diabetes should not be viewed as a single risk factor, but rather as a constellation of patient-specific factors, including admission glucose level, diabetes-related complications, and organ dysfunction. In particular, acute kidney injury and dysglycemia may have a greater impact on sepsis outcomes than the diagnosis of T2DM itself. These results suggest that risk assessment should be tailored to each patient with sepsis, particularly those with metabolic disease.

Our findings are in line with earlier meta-analyses which found no clear association or mixed results between diabetes and mortality in sepsis [11,12]. Narrative reviews have also proposed that the impact of diabetes on sepsis outcomes may be confounded by several factors such as severity of illness, source of infection, comorbid conditions, and treatment strategies [10].

Differences in outcomes between studies may also be due to differences in treatment exposure. The use of metformin and other glucose-lowering drugs in sepsis has been studied in several trials, but the results have been conflicting [30-34]. These differences indicate that the use of medication before or during hospitalization may contribute to the discrepancies in mortality estimates found in the literature.

Limitations

There are some limitations of this review. First, the main meta-analysis comprised only three studies, and the pooled estimate may be less precise and less powerful. Second, there was significant heterogeneity, suggesting that there was substantial variation between the studies included. Third, most studies were observational, and thus prone to residual confounding, selection bias, and differences in adjustment methods. Direct comparison between studies was also hampered by variations in the definition of diabetes, outcome measures, and effect estimates reported. Furthermore, this review was not prospectively registered, and Embase was not searched due to its unavailability. Future studies should not only consider diabetes as a binary exposure but should also investigate the clinical heterogeneity of T2DM. Multicenter prospective studies are required to assess the impact of glycemic control, diabetes duration, burden of complications, organ dysfunction, and treatment exposure on sepsis outcomes. Comparability across studies would be enhanced by standardized outcome reporting and predefined subgroup analyses. Meta-analyses of individual participant data may also be useful to help explain the sources of heterogeneity in the current evidence.

## Conclusions

This systematic review and meta-analysis found that the current available evidence does not demonstrate a statistically significant association between pre-existing T2DM and mortality among adults with sepsis. However, these findings should be interpreted with caution due to the limited number of studies included in the primary meta-analysis, the observational nature of the available evidence, and the substantial heterogeneity across studies. Emerging evidence suggests that factors such as glycemic control, diabetes-related complications, organ dysfunction, and antidiabetic treatment exposure may have a greater influence on sepsis outcomes than the presence of T2DM alone. Further prospective, well-adjusted, multicenter studies are needed to clarify the relationship between T2DM and sepsis outcomes and to better account for clinical heterogeneity.
